# Regulation of Endothelial-Specific Transgene Expression by the *LacI* Repressor Protein *In Vivo*

**DOI:** 10.1371/journal.pone.0095980

**Published:** 2014-04-22

**Authors:** Susan K. Morton, Daniel J. Chaston, Brett K. Baillie, Caryl E. Hill, Klaus I. Matthaei

**Affiliations:** 1 Blood Vessel Laboratory, The John Curtin School of Medical Research, The Australian National University, Canberra, Australia; 2 Stem Cell & Gene Targeting Laboratory, The John Curtin School of Medical Research, The Australian National University, Canberra, Australia; Brigham and Women's Hospital, United States of America

## Abstract

Genetically modified mice have played an important part in elucidating gene function *in vivo*. However, conclusions from transgenic studies may be compromised by complications arising from the site of transgene integration into the genome and, in inducible systems, the non-innocuous nature of inducer molecules. The aim of the present study was to use the vascular system to validate a technique based on the bacterial *lac* operon system, in which transgene expression can be repressed and de-repressed by an innocuous lactose analogue, IPTG. We have modified an endothelium specific promoter (*TIE2*) with synthetic *Lac*O sequences and made transgenic mouse lines with this modified promoter driving expression of mutant forms of connexin40 and an independently translated reporter, EGFP. We show that tissue specificity of this modified promoter is retained in the vasculature of transgenic mice in spite of the presence of *Lac*O sequences, and that transgene expression is uniform throughout the endothelium of a range of adult systemic and cerebral arteries and arterioles. Moreover, transgene expression can be consistently down-regulated by crossing the transgenic mice with mice expressing an inhibitor protein *LacI^R^*, and in one transgenic line, transgene expression could be de-repressed rapidly by the innocuous inducer, IPTG. We conclude that the modified bacterial *lac* operon system can be used successfully to validate transgenic phenotypes through a simple breeding schedule with mice homozygous for the *LacI^R^* protein.

## Introduction

For several decades, genetically modified mice have been the premium tool for the study of gene function *in vivo*. However, interpretation of the specific role of candidate genes can often be compromised in transgenic studies by the random nature of the site of transgene insertion into the host DNA [1], [2]. This non-specific “position effect” can result from transcriptional regulation imposed on the transgene by the adjacent host sequences, or alternatively, from the loss of function of genes which are accidentally interrupted by insertion of the transgene [1], [2].

Attempts to overcome these problems have employed inducible systems, such as the Cre-LoxP or Doxycycline-controlled methods, in which expression of the transgene can be regulated at specific times by an inducer molecule. Thus, phenotypes due to the transgene are isolated from effects due simply to the site of genetic insertion (for reviews see [3], [4]). However, both of these systems employ non-innocuous inducers for the switching of gene expression. The inducible CreERT system requires tamoxifen and the Doxycycline system utilises the antibiotic tetracycline, both of which can have off-target effects (reviewed in [4]). Tamoxifen, in particular, as a selective modulator of both membrane bound and intracellular estrogen receptors, is capable of altering vascular tone and blood pressure [5]; effects which are not conducive with uncovering a role for specific genes in cardiovascular function.

With the intention of creating an alternative system which utilises an innocuous inducer, Scrable and colleagues have modified the bacterial *lac* operator system for use in mammalian systems and reported reversible control of the tyrosinase gene and eye pigmentation, as well as the Huntington promoter driving a luciferase reporter, using the lactose analogue, Isopropyl β-D-1-thiogalactopyranoside (IPTG) [6]–[8]. Their system relied on the introduction into the tyrosinase and Huntington promoters of *Lac* operon (*Lac*O) sequences at specific distances spanning the transcription start site (see also [3]); a procedure which did not interfere with promoter function and specificity. However, in the presence of the LacI repressor protein (*LacI^R^*), the function of the promoter was repressed due to the binding of the *LacI^R^* protein to the *Lac*O sequences thereby blocking transcription. Following addition of IPTG, the *LacI^R^* protein was modified by the IPTG to undergo a conformational change that weakened its ability to bind to the *Lac*O sequences, thus permitting transcription factors to again bind normally and reinstate transcription. In this manner, gene function was controlled in a reversible manner using an agent that had no deleterious effect even after 8 months of continuous administration [6]. Surprisingly, only one report has appeared in the intervening decade, and this investigation, which utilised the haematopoietic system, failed to confirm that the *LacI^R^*/*Lac*O system could tightly and reversibly control gene expression [9]. However, results of this study were complicated by mosaic transgene expression amongst littermates within the same transgenic line [9]; effects likely due to the use of F2 progeny, in which multiple transgene insertion sites still existed. Furthermore, experiments in which IPTG was unable to reinstate transgene expression *in vivo* employed only a single bolus exposure to IPTG [9].

In the present study, we have re-evaluated the *LacI^R^*/*Lac*O system in the vasculature using transgenic mouse strains in which transgene inheritance had stabilised. Our gene of interest was the endothelial gap junctional protein, connexin40 (Cx40), for which identification of a role in the control of vascular function and blood pressure has been complicated by co-ordinate regulation of other connexin family members (e.g. [10]). We have created transgenic mouse lines that express mutant forms of Cx40 and an independently translated reporter, EGFP, selectively in the endothelium, through use of the endothelial specific promoter *TIE2* and its enhancer [11].

We show that addition of *Lac*O sequences to the *TIE2* promoter does not interfere with uniform transgene expression in the endothelium of a range of adult systemic and cerebral arteries and arterioles and that repression of promoter activity occurs reliably in the presence of *LacI^R^*, although the capacity for re-activation by daily administration of IPTG appears to vary with transgenic line. Nevertheless, the robust tissue-specific expression of transgenes directed by promoters modified with *Lac*O sequences, combined with the ability to significantly repress transgene function through breeding to mice ubiquitously expressing *LacI^R^*, makes this an attractive system to validate vascular phenotypes, ruling out “position effects” due to random transgene insertion.

## Materials and Methods

### Ethics Statement

All experimental animal procedures were carried out in strict accordance with the recommendations in the Australian code of practice for the care and use of animals for scientific purposes of the National Health and Medical Research Council of Australia. Protocols were approved by the Animal Experimentation Ethics Committee of the Australian National University (JMB.33.07; A2011/72).

### Generation of *TIE2Lac*O-Cx40-EGFP Constructs

**Figure 1:**
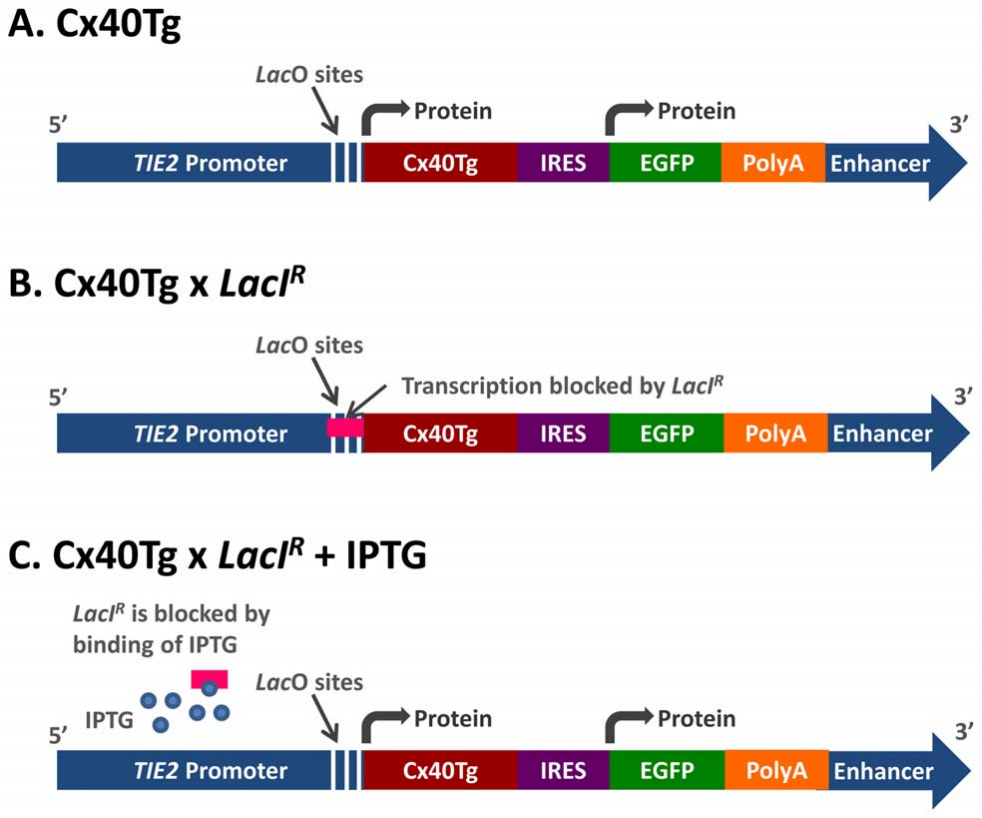
Control of transgene function by the *Lac*O/*LacI^R^* system. (A) Construct used to create Cx40 transgenic mice consists of a modified *TIE2* promoter containing *Lac*O transcriptional regulation sites (positions −117, −25 and +67), Cx40 transgene (Cx40Tg), an internal ribosomal entry sequence (IRES), EGFP, PolyA tail and *TIE2* Enhancer element. Proteins expressed from this construct are translated separately due to the presence of the IRES sequence. (B) When bred with *LacI^R^* mice, expression of the Cx40Tg should be repressed due to binding of the *LacI^R^* protein (pink) to the *Lac*O sites incorporated into the *TIE2* promoter. (C) In the presence of IPTG (blue circles) *LacI^R^* undergoes a conformational change lessening its affinity for the *Lac*O sites and transcription can proceed.

Constructs were created in which Cx40 and Enhanced Green Fluorescent Protein (EGFP) could be expressed separately through the use of an internal ribosome entry site (IRES) which allows the expression of a bicistronic mRNA and production of two independent proteins from the same promoter. This construct was coupled to the endothelial specific promoter, *TIE2*, as described below. The transgenic constructs were composed, in 5′ to 3′ order, of a modified *TIE2* promoter, the coding region of Cx40 with single base mutation, an internal ribosomal entry sequence (IRES), EGFP, human growth hormone poly A tail and a *TIE2* enhancer fragment (Figure 1A). Two different single base mutations encoding amino acids at positions 152 (T152A) and 202 (T202S; [12]) were incorporated into separate constructs.

*Lac*O sites were introduced into the *TIE2* promoter to enable regulation of transgene expression. The *TIE2* enhancer fragment was necessary for endothelial expression of the *TIE2* promoter throughout embryogenesis and adulthood [11].

#### *TIE2Lac*O construct

The plasmid pHHSDKXK [11], containing the *TIE2* promoter and a minimal enhancer fragment, as well as the plasmid pg50–2.11 containing the full length *TIE2* enhancer, were generous gifts from Professor Tom Sato, The University of Texas, Southwestern Medical Center at Dallas, Texas, USA. A 2 kb *TIE2* promoter fragment was recovered from pHHSDKXK by Hind III digestion. This was inserted into pBluescript KS II (pBKSII) by restricting with Hind III and Bam HI, ligating the insert to the vector Hind III site, blunting the vector BamH I and the fragment Hind III sites, and religating (with loss of the BamH I restriction site) to give pBKSII*TIE2*. The correct orientation of the *TIE2* promoter was determined by sequencing.

A BamH I – Nae I fragment was removed from this plasmid and replaced with a synthetic fragment of the *TIE2* promoter now containing *Lac*O sites. This synthetic BamH I-Nae I *Lac*O fragment, pUC57*TIE2Lac*O (synthesised by Genscript, USA) was designed with three 18 bp *Lac*O sites (5′-ATT-GTG-AGC-GCT-CAC-AAT-3′), that were introduced to span the transcription start site, with 92 bp intervals, at positions –117, −25 and +67 of the *TIE2* promoter. These locations were determined not to contain known transcription factor binding sites (MatInspector). The resulting construct, *TIE2Lac*O, contained the full length *TIE2* promoter with 3 *Lac*O sites.

#### *TIE2Lac*O-Cx40-EGFP construct

Digestion of *TIE2Lac*O with Cla I, followed by blunting of the Cla I site, and partial digestion with Bcl I allowed recovery of the 2.2 kb *TIE2Lac*O fragment. This was ligated into the Sac II digested, blunted, then BamH I-digested 7.7 kb backbone fragment of pBKSII containing the Cx40-IRES-EGFP construct.

The *TIE2Lac*O-Cx40-EGFP plasmid was linearised by digestion with Xho I, blunted, and ligated to the 10 kb *TIE2* enhancer fragment from Nae I and Sal I-digestion of pg50–2.11. The final construct contained the enhancer in the forward orientation after the polyadenylation signal (Figure 1A). Sequencing was performed at each step to confirm the presence, fidelity, and orientation of construct components.

### Generation of transgenic mice

#### *TIE2Lac*O-Cx40Tg-EGFP mice

The final construct was digested with Pvu I and BssH II and gel purified to remove vector sequence prior to use in the generation of transgenic mice. Transgenic C57BL/6 mice were produced by pronuclear injection of the 19 kb construct (TASQ, University of Queensland, Australia). Transgenic founders were identified by PCR.

#### Selection of transgenic lines

Copy number of the transgene was determined in founder mice (N0) and offspring of the next three generations (N1-N3), using quantitative real time PCR (qPCR) for the reporter gene EGFP relative to a known single copy gene, transferrin. EGFP expression was quantified using Western Blotting. Mice with different transgene copy numbers were bred as separate colonies from N1, N2 and N3 generations to confirm segregation and stabilisation of different copy numbers.

#### LacIR mice

Mice from this strain were developed to be homozygous for a transgene that expresses the *LacI^R^* inhibitor protein under control of the ubiquitous β-actin promoter [8]. The heterozygous *LacI^R^* strain was a generous gift from Professor Heidi Scrable, University of Virginia, Charlottesville, Virginia, USA. Homozygous mice were identified from heterozygous matings using qPCR of genomic DNA for *LacI^R^.* Homozygosity was determined by test breeding with wildtype mice and by quantifying expression of *LacI^R^* protein in offspring by Western Blotting.

*TIE2LacO-Cx40Tg-EGFP/LacI^R^ mice* were generated by crossing the *TIE2Lac*O-Cx40Tg-EGFP mice with homozygous *LacI^R^* mice (Figure 1B) [8].

### Quantitative Real Time PCR (qPCR)

#### qPCR to determine EGFP Transgene Copy Number

Two standard reference curves were generated. The first was a series of 2-fold dilutions of wildtype mouse genomic DNA, from 8 ng/µl to 0.25 ng/µl, in T_10_E_0.1_ containing 4 ng/µl tRNA (Sigma) as carrier. The mouse transferrin gene was used as a single copy reference [13] using the primers detailed in Table 1. The second standard used purified plasmid DNA containing the entire *TIE2Lac*O-Cx40-EGFP transgene construct. Serial 10-fold dilutions of the plasmid DNA were made from 2.5×10^−2^ ng/µl to 2.5×10^−7^ ng/µl, again using T_10_E_0.1_ containing 4 ng/µl tRNA as carrier. The primers for EGFP are detailed in Table 1.

**Table 1:** Primer sequences and conditions for quantitative PCR.

Primer	Sequence	Annealing Temp	Amplicon size
Transferrin F	5′-ATCCCAGTGACTACAACGGTTCCA-3′	63°C	189bp
Transferrin R	5′-AGACCACAACATGGTTTGGAGCTT-3′		
EGFP F	5′-TCTATATCATGGCCGACAAGCAGA-3′	65°C	165bp
EGFP R	5′- ACTGGGTGCTCAGGTAGTGGTTGT 3′		
*LacI^R^* F	5′-GGTGTCTCTTATCAGACTGTTTCCA-3′	67°C	206bp
*LacI^R^* R	5′-GCTGCCACAATTTGAGATGGTGCA-3′		

Genomic DNA from each mouse to be tested was quantified using the high sensitivity QUBIT assay (Invitrogen) and diluted to 4 ng/µl in T_10_E_0.1_. Each experimental sample was prepared in triplicate and amplified for EGFP and transferrin. Each qPCR run contained the 6 standard reference samples for transferrin and for EGFP, 15 test samples and a negative control without DNA, all in triplicate.

Each qPCR reaction contained 10µl of 2x Sensimix+SYBR (No ROX) master mix (Bioline), 5µM forward and reverse primer, 5µl of DNA sample and water to a total volume of 20µl. PCR was performed in a Corbett Research Rotor-Gene RG-3000 Real Time Analyser with cycling conditions as follows: 95°C for 10 minutes, followed by 40 cycles of 95°C for 10 s, annealing temp (see Table 1) for 20 s and 72°C for 20 s. Melt curves were performed after each run to ensure the amplification of a single product. The data was analysed using the Rotagene 6.0 software and copy number calculated from the standard curves.

#### qPCR to determine *LacI* Zygosity

Genomic DNA from a mouse known to be heterozygous for the *LacI^R^* transgene was quantified using the QUBIT (Invitrogen) and diluted to 16 ng/µl in T_10_E_0.1_ containing 4 ng/µl tRNA as carrier. A standard curve ranging from 8 ng/µl to 0.25 ng/µl genomic DNA was prepared by 2-fold serial dilutions in triplicate in T_10_E_0.1_ containing 4 ng/µl tRNA as carrier diluent. Transferrin was again used as a single copy gene reference [13].

DNA samples from test mice were prepared as above in triplicate and amplified with the *LacI^R^* and transferrin primers as detailed in Table 1. The ratio of *LacI^R^* to transferrin for the known heterozygote was normalised to 1.00. Test samples were normalised accordingly and the ratio used as a predictor of zygosity.

### Western blot detection of EGFP

Mice were anaesthetised with isoflurane, decapitated, and mesenteric arteries were removed and stripped of fat in ice cold phosphate buffered saline (PBS) prior to being snap frozen in liquid nitrogen. Frozen tissues were pulverised under liquid nitrogen in the presence of 100µl of frozen extraction buffer (37 mM TRIS, 0.5% lithium dodecyl sulphate, 2.5% glycerol, 0.13 mM EDTA, 0.06 mM SERVA blue G250, 0.04 mM phenol red, 50 mM DTT, 1X cOmplete mini Protease Inhibitor Cocktail [Roche], pH 8.5). Frozen extracts were allowed to thaw on ice and the resulting liquid extract was transferred to a plastic sample tube. The remaining sample was washed from the mortar using a further 50µl of sample buffer and recovered into the same plastic sample tube. Extracts were then heated at 70°C for 5 min, vortexed and heated for a further 5 min before being stored at −20°C until analysis.

Proteins present in tissue extracts were resolved by SDS denaturing electrophoresis on NuPAGE 4–12% BIS-TRIS gradient gels using NuPAGE MES buffer according to the manufacturer's recommended protocols (Invitrogen). Proteins were transferred onto PVDF membranes at 180 mA in NuPAGE transfer buffer for 4 h at RT.

Membranes were recovered, and air dried at room temperature for 1 h. Membranes were rehydrated with 100% methanol, followed by 50% methanol in PBS for 5 minutes each. Membranes were then blocked with 2% BSA in PBS for 1 hour at RT. Membranes were washed 6 times with PBST (PBS, 0.05% Tween-20) for 10 minutes each. Primary antibody: mouse anti-GFP monoclonal IgG1 (kindly supplied by Jan Elliot, Research School of Biology, Australian National University), mouse anti-*LacI* (Millipore), mouse anti-α-smooth muscle actin (αSMA, Sigma), rabbit anti-vonWillebrand Factor (vWBF, Dako); was applied to the blot (1∶1000 anti-GFP; 1∶2000 anti-LacI; 1∶10000 anti-αSMA; 1∶2000 anti-vWBF; diluted in 2% BSA in PBST) for 16 hrs at 4°C. Membranes were washed 6 times with PBST for 10 minutes. Secondary antibody (goat anti-mouse HRP conjugate, Millipore, or goat anti-rabbit HRP conjugate, Sigma) was applied to the blot (1∶2000 for EGFP; 1∶2000 for LacI; 1∶10000 for αSMA, 1∶2000 for vWBF; diluted in 2% BSA in PBST) for 2 h at RT. Membranes were washed 6 times in PBST, followed by 1 wash in PBS, each for 10 minutes. The signal was developed using Immobilon Western chemiluminescent substrate according to the manufacturer's recommended protocol (Millipore). Image recording was performed on a LAS1000 system (Fujifilm). The EGFP +ve control was from a mouse expressing an EGFP tagged with a nuclear localization signal and the EGFP protein therefore has a slightly higher molecular weight than standard EGFP.

Band signal intensity was measured using ImageQuant TL Software Version 7.0 (GE Healthcare). EGFP and LacI quantification was normalised to either the smooth muscle marker, α-actin, or the endothelial marker, vWBF expression, respectively.

### Immunohistochemical detection of EGFP

Mice were anaesthetised with isoflurane, decapitated and kidneys, mesenteric and basilar arteries removed. Kidneys were placed in ice cold PBS and the capsules were removed and the kidneys were cut into 3–4 mm transverse slices. Mesenteric arteries were placed into ice-cold PBS and surrounding fat removed. All tissues were immersion fixed in 2% paraformaldehyde and 0.01% sodium nitrite (100 mM sodium phosphate buffer) for 10 min, then cryprotected in 30% sucrose in PBS overnight. Tissues were finally embedded in Tissue Tek O.C.T (Sakura Fintek USA, Torrance, CA), frozen and cut into 30µm sections using a cryostat.

#### Cremaster arterioles

Cremaster muscles were removed from mice which were anaesthetised (1 mg/kg medetomidine, 10 mg/kg midazolam, and 0.1 mg/kg Fentanyl, i.p.) and continuously perfused with anaesthetic (0.2 mg/h medetomidine, 0.2 mg/h midazolam, 0.002 mg/h fentanyl) via a jugular vein cannula during the procedure. Isolated cremaster muscles were fixed in 2% paraformaldehyde and cryosections prepared as above. Mice were euthanised by cervical dislocation whilst still deeply anaesthetised.

Tissue sections were preincubated for 30 min in PBS containing 2% bovine serum albumin, 0.2% Triton-X100, and 0.04% sodium azide before being incubated at room temperature for 24 h with rabbit anti-GFP antibodies (Invitrogen) diluted 1∶1000 in the pre-incubation solution. Sections were washed three times in PBS and incubated for 2 h with Alexa-488 conjugated donkey anti-rabbit antibodies (Invitrogen) diluted 1∶400 with PBS containing 0.02% Triton-X100. Sections were then incubated in PBS containing 0.01% pontamine sky blue for 3 min to shift the green autofluorescence of the vascular internal elastic lamina to longer wavelengths. Finally, sections were washed three times with PBS before being mounted in buffered glycerol.

### Confocal microscopy and image analysis

Image series of all stained tissues were taken with a Leica SP2 confocal scanning microscope. For time course studies, the same acquisition settings were used to collect images to enable the degree of staining to be compared at different time points. For quantification of EGFP staining in the kidney, image series were taken using a 20x lens with *z plane* increments of 1.5 µm. For each image series, an average projection image was generated and three such composite images from each animal were used to quantify EGFP expression.

#### Quantification of EGFP staining

For each image, areas of endothelial tissue were identified by their location on the inner surface of the auto-fluorescence generated by the internal elastic lamina (red). Using the polygon selection and histogram functions in ImageJ (National Centre for Biotechnology Information), the intensity of EGFP staining (green) was calculated. For each image the EGFP signal within several non-endothelial tissues such as tubules and vascular smooth muscle were also calculated. EGFP expression was quantified as signal to noise ratio minus one using the equation, (Endothelial Cell Intensity/Background Intensity)-1.

### IPTG Treatment of *TIE2Lac*O-Cx40Tg-EGFP/*LacI^R^* mice

The *TIE2Lac*O-Cx40Tg-EGFP/*LacI^R^* mice were given IPTG (Inalco, USA; Figure 1C) with doses ranging from 10 to 80 mM in the drinking water for periods of 2 to 14 days. Sucrose (2%) was added to encourage consumption at higher concentrations (40 and 80 mM); an approach commonly used to reduce the bitter taste of Doxycycline [14]. Controls received 2% sucrose in water. Doses as high as 10% sucrose have previously been reported to have no effect on blood glucose, insulin or adiponectin levels, nor on glucose tolerance [15]. Following treatment, mice were euthanised and analysed for EGFP expression by Western Blotting or immunohistochemistry.

### Statistical Analysis

Data are presented as means ± SEM where n represents the number of mice. Statistical significance was determined by one-way ANOVA with Bonferonni post-hoc test for multiple comparisons, with *P*<0.05 considered significant.

## Results

### Transgene Inheritance is unstable in early generations

Transgene copy number was monitored in the T152ATg mouse strain over the first generation (N1) and found to vary from 4 to 22 copies. Subsequent breeding to C57BL/6 wildtype mice showed that this variability continued into the next two generations before stabilizing with breeder selection at 12 copies. The production of equal numbers of transgenic to wildtype offspring in each generation was consistent with a single integration site, suggesting some instability of the multiple transgene copies at the one insertion site.

The T202STg strain was bred for 6 generations to wildtype mice and copy number stabilized at 360 copies. At this stage, equal numbers of transgenic to wildtype offspring were still found in each generation, again consistent with a single integration site.

### Insertion of *Lac*O sites in the TIE2 promoter does not compromise endothelial specificity

In order to determine the cellular distribution of the transgene expression, we conducted immunohistochemical studies in the vasculature using antibodies against the reporter EGFP. We did not study Cx40 expression as antibodies against Cx40 were unable to discriminate the Cx40 transgene from wildtype Cx40, which is highly expressed in the vascular endothelium [16].

**Figure 2:**
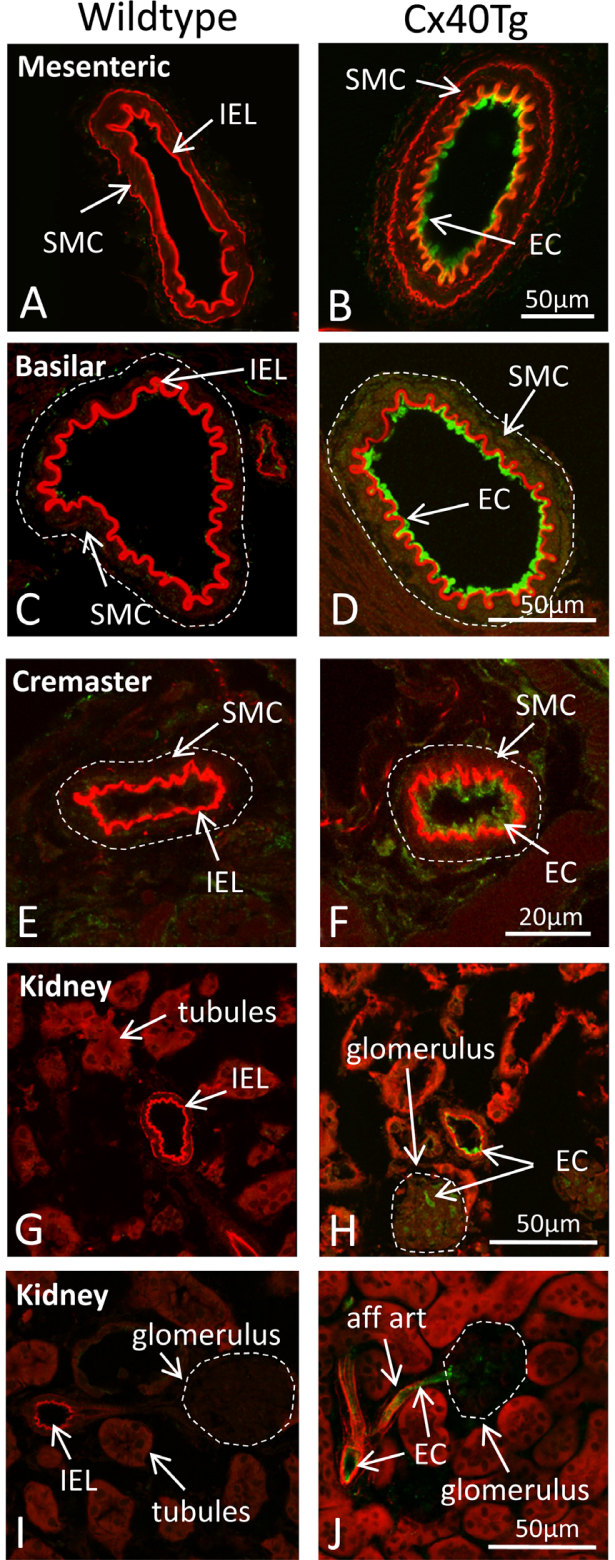
Maintenance of endothelial tissue specificity of the *TIE2* promoter when modified with *Lac*O sites. EGFP was expressed in the endothelial cells (EC) of primary mesenteric arteries and basilar arteries of transgenic mice (Cx40Tg; B, D), as well as in cremaster muscle arterioles (F) and kidney arteries, arterioles and glomerular capillaries (H, J). EGFP was not detectable in arterial smooth muscle (SMC) or in renal tubules. No EGFP was detected in comparable vessels of wildtype mice (A, C, E, G, I). IEL represents the internal elastic lamina; aff art, afferent arteriole. Examples from Cx40T152ATg (B, J) and Cx40T202STg mice (D, F, H).

No staining was detected in arteries or arterioles of wildtype mice (Figure 2A, C, E, G, I). In contrast, EGFP was detected uniformly in the endothelium of arteries and arterioles of several different systemic and cerebral circulations of transgenic mice, while staining was absent from the arterial smooth muscle or adventitia. Vessels studied included mesenteric (Figure 2B) and basilar (Figure 2D) arteries, as well as cremaster muscle arterioles (Figure 2F), renal arteries, arterioles and glomerular capillaries (Figure 2H, J).

### Homozygous *LacI^R^* mice can be reliably identified by qPCR

Since repression of transgene expression required interbreeding of transgenic mouse strains (*TIE2Lac*O-Cx40Tg-EGFP mice and *LacI^R^* mice), we developed a homozygous *LacI^R^* strain to ensure that all offspring in each generation would express *LacI^R^* and 50% of these mice would also be transgenic for Cx40. Our first aim was therefore to develop a rapid assay to detect homozygous offspring from heterozygous matings and validate the assay by test breeding and Western blot protein assay.

**Figure 3:**
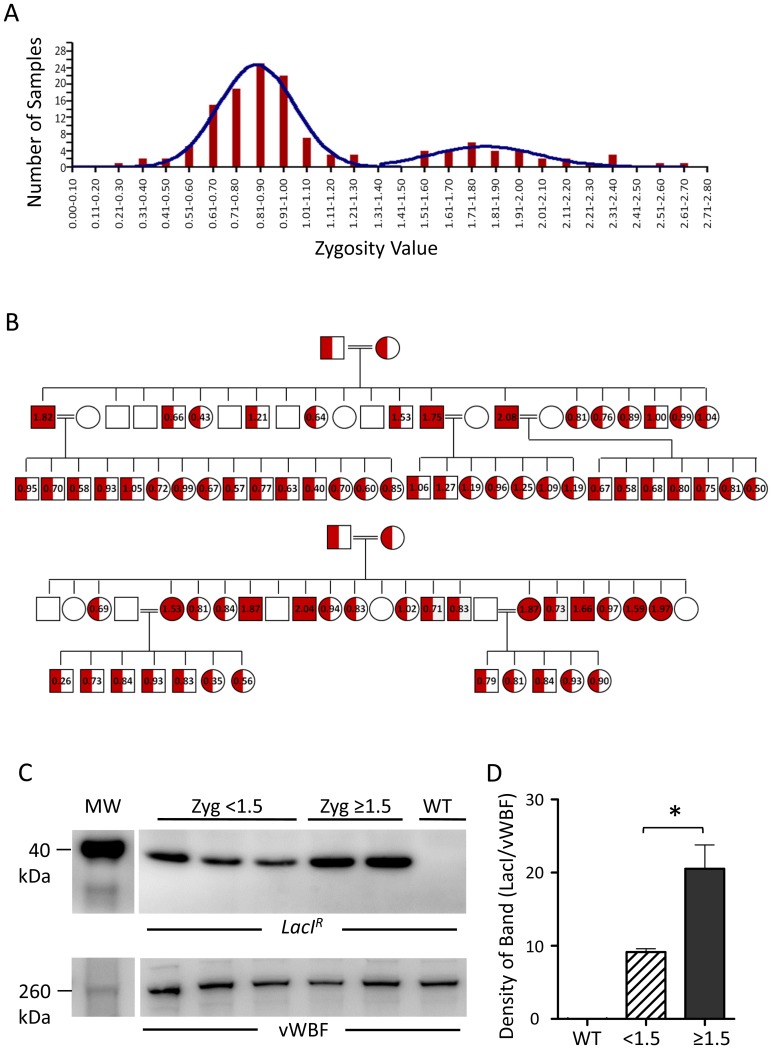
Detection of *LacI^R^* homozygosity using quantitative PCR. (A) Binning of zygosity values from heterozygous matings demonstrates two populations indicative of heterozygotes and homozygotes. (B) Interbreeding of predicted heterozygotes produces wildtype, heterozygote and homozygote populations in Mendelian ratios. (C) *LacI^R^* expression in predicted homozygotes (zygosity ≥1.5) is greater than in predicted heterozygotes (zygosity <1.5). Representative blots and group data for 3 heterozygote and 5 homozygote samples. WT wildtype. *P<0.05 denotes significant difference.

Offspring from matings of heterozygous *LacI^R^* mice were used to provide samples for qPCR using primers and standard curves for both *LacI^R^* and the single copy gene, transferrin. *LacI^R^*/transferrin values were calculated for each mouse and zygosity values obtained by normalizing these values to that of a known heterozygote *LacI^R^* mouse, whose zygosity was defined as 1.0. When data from 136 offspring were plotted, a bimodal distribution was found, consistent with a large heterozygous and smaller homozygous population (Figure 3A). From this distribution we defined a homozygote as any animal with a zygosity value ≥1.5.

Figure 3B shows that interbreeding of 4 heterozygous mice, defined by zygosity values <1.5, produced a total of 42 offspring of which 11 were wildtype (zygosity  = 0), 21 were heterozygotes (zygosity <1.5) and 10 were homozygotes (zygosity ≥1.5). These numbers accord well with Mendelian segregation which would predict a ratio of 10.5 wildtype: 21 heterozygotes: 10.5 homozygotes from a heterozygous mating producing the same number of offspring.

Subsequent breeding of 5 mice with zygosity values ≥1.5 (1.5, 1.8, 1.8 1.9, 2.1) to wildtype mice, each produced 100% *LacI^R^* transgenic mice (41 offspring), with zygosity values <1.5 (Figure 3B), as expected for a homozygous/wildtype mating.

Western blotting showed that mesenteric arterial samples taken from mice with zygosity values ≥1.5 showed higher *LacI^R^* expression than samples from mice with zygosity values <1.5 (Figure 3C). Quantification of *LacI^R^* bands, relative to the endothelial marker vWBF, showed that *LacI^R^* expression was 2-fold higher in samples from mice with zygosity values of ≥1.5 compared to those from mice with zygosity values of <1.5 (Figure 3D), consistent with assignation of homozygosity and heterozygosity to zygosity values of ≥1.5 and <1.5, respectively.

### *LacI^R^* protein significantly reduces transgene expression in vivo

**Figure 4:**
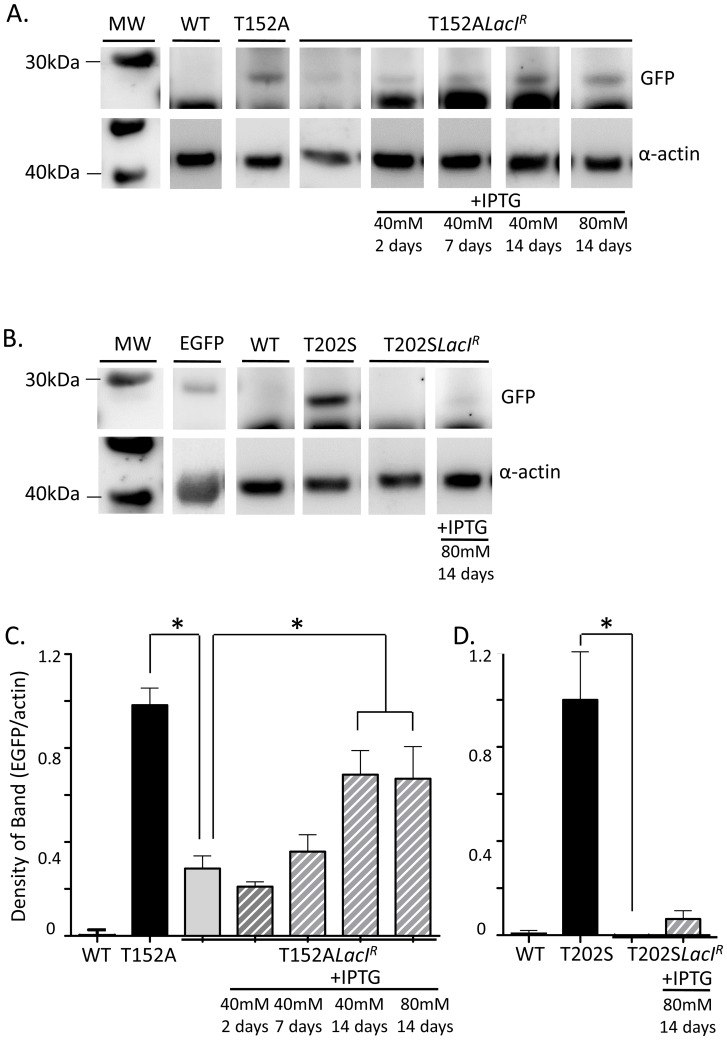
Control of EGFP expression by *LacI^R^* and IPTG: Western blotting. Western blots of mesenteric arterial samples demonstrate repression of EGFP expression by breeding of Cx40 (T152ATg and T202STg) transgenic mice with *LacI^R^* mice (T152A*LacI^R^* mice: A, C; T202S*LacI^R^* mice: B, D). De-repression of EGFP by IPTG is found in T152A*LacI^R^*mice after 14 days (A, C), but not in T202S*LacI^R^* mice (B, D). (C). Group data (left to right): n = 6, 9, 11, 5, 5, 8, 7 mice. (D). Group data (left to right): n = 4, 6, 2, 4 mice. WT, wildtype. Positive control for EGFP is from a mouse expressing EGFP tagged with a nuclear localisation signal and therefore has a slightly higher molecular weight. *P<0.05 denotes significant difference; ANOVA followed by Bonferonni tests for multiple groups.

When *TIE2Lac*O-Cx40Tg-EGFP mice were interbred with *LacI^R^* mice, EGFP expression in primary mesenteric arteries of both transgenic strains, T152A*LacI^R^* and T202S*LacI^R^*, was reduced significantly by 71% and 100%, respectively (Western blots; T152A*LacI^R^*: Figure 4A, C; T202S*LacI^R^*: Figure 4B, D).

**Figure 5:**
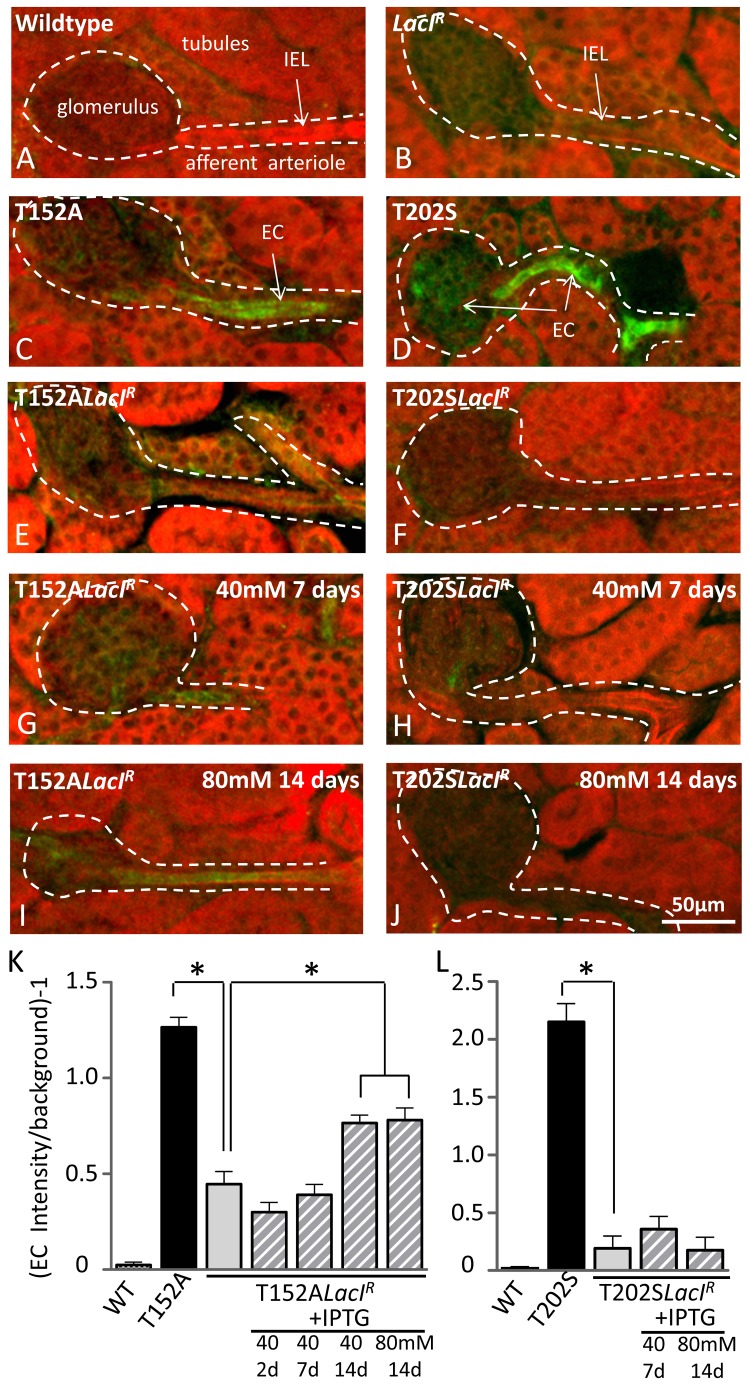
Control of EGFP expression by *LacI^R^* and IPTG: Immunohistochemistry. Immunohistochemical staining for EGFP demonstrates endothelial (EC) staining in T152A (C) and T202S transgenic mice (D) but not in wildtype (A) or *LacI^R^* (B) mice. Interbreeding of transgenic mice with *LacI^R^* mice led to a significant reduction in EGFP staining in both transgenic strains (E, F). De-repression of EGFP by IPTG is found in T152A*LacI^R^* mice after 14 days (I, K), but not in T202S*LacI^R^* mice (J, L). (K) Group data (left to right): n = 7, 12, 7, 4, 5, 7, 5 mice. (L) Group data (left to right): n = 6, 8, 4, 8, 4 mice. *P<0.05 denotes significant difference; ANOVA followed by Bonferonni tests for multiple groups.

Immunohistochemical analyses of kidney sections of T152A*LacI^R^* and T202S*LacI^R^* mice showed that endothelial specific expression of EGFP was similarly reduced by 65% and 91%, compared to T152ATg and T202STg mice, respectively (T152A*LacI^R^*: Figure 5A, C, E, K; T202S*LacI^R^*: Figure 5B, D, F, L).

Expression of *LacI^R^* protein was not significantly different between mesenteric arterial samples taken from T152A*LacI^R^* and T202S*LacI^R^* mice, when quantified relative to the smooth muscle marker, α-actin (T152A*LacI^R^* mice: 3.2±0.5 *LacI^R^/*actin, n = 9 mice; T202S*LacI^R^* mice: 3.1±0.3 *LacI^R^/*actin, n = 6 mice).

Together, these data show that the *LacI^R^* protein is expressed to the same extent in the 2 transgenic mouse strains and can reliably and significantly reduce transgene expression *in vivo.*

### De-repression of transgene expression can be achieved by administration of IPTG

Preliminary experiments testing low concentrations of IPTG (10 and 20 mM) produced variable induction of transgene expression (data not shown). To encourage consumption of higher concentrations of IPTG (40 and 80 mM), 2% sucrose was added to the drinking water. Measurement of blood glucose before and after 2% sucrose treatment confirmed that blood glucose levels were not altered by sucrose addition for 7 or 14 days (Before: 7.8±0.4 mM, n = 8 mice; 7 day sucrose: 8.1±0.3 mM, n = 8 mice; 14 day sucrose: 6.8±0.3 mM, n = 8 mice). Addition of either 40 mM or 80 mM IPTG with 2% sucrose to the drinking water did also not significantly alter the average volume of water drunk by each mouse per day (2% sucrose in water: 4.5±0.6 ml, n = 6 mice; 40 mM IPTG+ sucrose: 4.6±0.5 ml, n = 9 mice; 80 mM IPTG+ sucrose: 5.1±0.5 ml, n = 12 mice). Mice given IPTG did not show any abnormalities in regard to coat appearance, hydration, grooming, general activity, or food and water consumption.

Administration of 40 mM IPTG to T152A*LacI^R^*mice over a period of 14 days led to a gradual increase in transgene expression in mesenteric arteries, as measured by Western blotting (Figure 4A, C). After 14 days of IPTG treatment, transgene expression was significantly higher than in untreated T152A*LacI^R^* mice and had recovered to 70% of that in T152A transgenic mice (Figure 4C). When assayed immunohistochemically in kidney sections, endothelial transgene expression similarly increased over 14 days, with expression after 14 days' treatment significantly increased compared to untreated T152A*LacI^R^*mice and recovery to 60% of that in T152A transgenic mice (Figure 5E, G, K). Doubling the concentration of IPTG to 80 mM did not produce any further increase in transgene expression after 14 days' treatment (Western blot: Figure 4A, C; Immunohistochemistry: Figure 5I, K).

In contrast, administration of 40 mM or 80 mM IPTG to T202S*LacI^R^* mice for 14 days failed to significantly induce transgene expression, assayed either by Western blotting (Figure 4B, D) or immunohistochemistry (Figure 5F, H, J, L). Moreover, the minimally increased transgene expression that was seen in T202S*LacI^R^* mice, not only varied between animals, but also amongst vessels in the same animal (data not shown).

## Discussion

The present study has tested a modified bacterial *lac* operon system, developed originally by Scrable and colleagues [6]–[8], to reversibly control gene expression in the vasculature *in vivo.* We show that this system can be used to reliably repress gene expression selectively in the vascular endothelium, through the use of the modified endothelial specific promoter *TIE2* [11]. This ability thus enables the unambiguous attribution of phenotype to the transgene, eliminating any influence of the site of transgene integration into the genome. Our data also show that transgene expression can be de-repressed rapidly by the innocuous inducer, IPTG, in one transgenic line.

### Modification of the promoter with *Lac*O sites

Modification of the *TIE2* promoter with three *Lac*O sites and generation of transgenic mice did not alter the tissue specificity of this promoter in directing expression of the EGFP reporter. The uniform, endothelial cell specific protein expression for EGFP found here in several different arteries and arterioles of adult mice was entirely consistent with the expression pattern reported in the literature for the *TIE2* promoter with its enhancer element [11]. These data thus extend the findings of Scrable and colleagues in the use of this system, from more generalised promoters [6]–[8] to a vascular specific promoter, to show that tissue specificity is not compromised by the addition of three *Lac*O sequences spanning the transcription start site. However, although our data suggests that a large number of different promoters might be amenable to such a modification, their specificity will have to be carefully verified after introduction of the *Lac*O sites.

In contrast to the uniform transgene expression that we found with the *TIE2* promoter, Grespi and colleagues reported variable expression amongst different immune cell populations and different tissues when directed by the *Vav-gene* promoter [9]. While some of this variegation may have resulted from the persistence of multiple transgene insertion sites in F2 progeny, it should also be noted that the *Vav-gene* construct contained viral sequences, both in the intron, as well as the PolyA signal. Mosaic expression might therefore be expected as these sequences would be subjected to silencing through methylation. It is possible that our constructs avoided such modifications since they consisted entirely of mammalian DNA sequences.

### Silencing of transgene expression in vivo with *LacI^R^*

Expression of EGFP in both of the transgenic mouse lines established in this study was significantly repressed by interbreeding with a mouse line ubiquitously expressing the repressor protein *LacI^R^.* In T202S*LacI^R^* mice, expression was fully repressed, while in T152A*LacI^R^* mice, transgene expression was repressed by 70%. Our current studies of cardiovascular function have confirmed that repression in both transgenic lines is extended in a similar manner to the physiological phenotypes resulting from expression of the mutant endothelial Cx40 transgenes. We see altered blood pressure which is significantly reversed by *LacI^R^* in both strains of mice (data under Journal submission). Such repression of transgene expression by *LacI^R^ in vivo* enables the unequivocal attribution of phenotypes to transgenes, thus eliminating artefacts due to the site of integration into the genome. The development and validation in this study of a simple and reliable method for identifying homozygous transgenic *LacI^R^* mice using qPCR, further facilitates the use of this system by increasing the probability of production of the requisite doubly transgenic mice.

### De-repression of transgene expression in vivo using IPTG

Of surprise was the finding that the ability of IPTG to reinstate transgene expression varied between the 2 transgenic mouse strains. Thus, in T152A*LacI^R^* mice, daily administration of IPTG led to a rapid reinstatement of transgene expression to 70% of full activity, while in the T202S*LacI^R^* mice, transgene expression was variable and inconsistent. A correlation thus existed between the extent of repression by the repressor protein *LacI^R^* and the ability of IPTG to reinstate transgene expression. The variation in transgene regulation was unexpected, as the T152A*LacI^R^* and T202S*LacI^R^* transgenic mice were produced using the same promoter construct with the three *Lac*O sites located in the same positions. Indeed, the only differences in the constructs were the two, single nucleotide changes within the connexin gene sequence: a change unlikely to affect the ability of a promoter to regulate transgene expression.

The variability in de-repression *in vivo* of the T152A and T202S transgenes suggests that the activity of the *TIE2Lac*O promoter may have been affected by the site of transgene integration. However, this is unlikely since the use of the *TIE2* enhancer sequences, in conjunction with the *TIE2* promoter, has been shown to ensure consistent and endothelial cell specific activity [11], [17], [18], as we confirm here. Differences in the availability of the *LacI^R^* protein to the *Lac*O sequences also seem unlikely, since *LacI^R^* protein expression was identical in mesenteric arteries of the T152A*LacI^R^* and T202S*LacI^R^* transgenic mice. Finally, variability in access and activity of IPTG in the target endothelial cells is unlikely, as IPTG was replenished daily over the 14 day exposure period and previous studies have shown that IPTG can remain active after crossing both the blood-brain barrier and the placenta [8].

In spite of the similarity in the 2 transgenic constructs, a major difference that did exist between the T152A and T202S transgenic mouse strains was transgene copy number. Interestingly, the high copy number strain (T202S) was the one in which transgene expression could be completely repressed by *LacI^R^* but not de-repressed by IPTG. Since it is known that high copy number transgenic mice have more highly compacted heterochromatin (see for e.g.[19], [20]) and that the *LacI^R^* protein forms tetramers between a number of different *Lac*O sites [21], it is possible that the numerous T202S transgene copies are more tightly packed and more compressed by *LacI^R^*, than the less numerous T152A transgene copies. It would then follow that this conformation could not be de-repressed by IPTG. Indeed if correct, this phenomenon could be an advantage for future studies where different founders could be chosen based on copy number, allowing a gene to be totally repressed in high copy number founders, and de-repressed in low copy number founders. However, more founder lines with varying copy numbers are required to confirm this association.

## Conclusions

Using a vascular cell type specific promoter, we have shown that the repressor protein *LacI^R^* of the bacterial *lac* operon system, when modified for use in mammalian cells, can reliably repress transgene expression *in vivo*, thus enabling the attribution of phenotype to the transgene, rather than to the insertion site. Moreover, the introduction into the promoter of *Lac*O sites does not compromise tissue specificity, making this an attractive system to validate transgenic phenotypes. While rapid de-repression of transgene expression by the innocuous inducer, IPTG, has been reported previously for both the tyrosinase and Huntington promoters [6]–[8], in the present study this only occurred reliably in one of our transgenic lines, even though the constructs were essentially identical. Should studies in which de-repression be of interest, for example, to study gene expression in adulthood without developmental complications, the development of several founders may be required to identify a line in which this feature of the system is possible. In this connection, the inverse correlation between the degree of repression by *LacI^R^* and the ability of IPTG to reinstate expression, a feature also described in a previous study [9], may be a useful index to rapidly identify transgenic lines in which de-repression by IPTG will be possible.
